# Optimized haemostasis in nephron-sparing surgery using small-intestine submucosa

**DOI:** 10.1186/1471-2490-8-8

**Published:** 2008-04-29

**Authors:** Jörg Simon, Robert de Petriconi, Michael Meilinger, Richard E Hautmann, Georg Bartsch

**Affiliations:** 1 Department of Urology, University of Ulm, Prittwitzstr. 43, 89075 Ulm, Germany

## Abstract

**Background:**

The indications for nephron-sparing surgery are expanding constantly. One major contributing fact for this development is the improvement of haemostatic techniques following excision of the tumor. Nevertheless, postoperative bleeding complications still occur. To prevent this, we prospectively studied the effect of application of small-intestine submucosa (SIS) over the renal defect.

**Methods:**

We performed 55 nephron-sparing surgeries applying SIS between 08/03 and 10/06 in 53 pts. (mean age: 59 yrs., range 29 – 79 yrs.). After resection of the renal tumor and application of a haemostyptic agent, we used SIS to secure and apply compression on the defect.

**Results:**

The final pathology revealed clear-cell and papillary carcinoma, papillary adenoma, oncocytoma, and angiomyolipoma in 39 (70.9%), 6 (10.9), 1 (1.8%), 2 (3.6%) and 7 (12.7%) patients, respectively. The 45 malignant lesions (81.8%) were classified as pT1a and pT1b in 35 (77.8%) and 10 (22.2%) patients, respectively. The median tumor size was 4.5 cm (range: 1.3 – 13 cm). The median operating time was 186 min (range: 90 – 260 min). 18 (32.7%) procedures were performed without ischemia. 23 (41.8%) and 14 (25.5%) cases were operated in in-situ cold and warm ischemia, respectively. The median intraoperative blood loss was 730 cc (range: 100 – 2500 cc). No open operative revision was indicated due to postoperative bleeding complications. Furthermore, there was no necessity to substitute persistent blood loss from the drains postoperatively. No urinoma occurred.

**Conclusion:**

SIS is a highly effective and easy-to-use instrument for preventing postoperative bleeding and urinary fistula complications in nephron-sparing surgery.

## Background

Nephron-sparing surgery provides effective therapy for patients that require preservation of renal function [1]. Furthermore, due to the low recurrence rates and excellent survival rates of nephron-sparing surgery in patients with renal tumors 4 cm or smaller, nephron-sparing surgery has become an accepted alternative treatment for patients with normal renal function [1].

The reported major complications after in-situ nephron-sparing surgery for solid renal lesions are urinary fistulas (2.1% to 17.4%), infections/abscesses (0.6% to 6.0%) and bleedings (0% – 7.9%), resulting in an overall re-operation rate of 0% to 3.1% [1]. Several technical considerations to prevent these complications have been propagated: watertight closure of the collecting system, rigorous ligation of vessels and reconstruction of the renal remnant over a haemostatic agent [1]. These principles resulted in a dramatic decrease of the complication rates following nephron-sparing surgery in the last decades [2,3].

Porcine small-intestine submucosa (SIS, Surgisis^® ^Soft Tissue Graft, COOK Biotech, West-Lafayette, Ind, USA) is a natural acellular biomaterial based on collagen from the porcine submucosa. SIS does not induce major adverse reactions when surgically implanted and is gradually remodeled, leaving behind autologous tissue. The membrane is used for different purposes in urology, obstetrics and gynaecology, general surgery and wound care [4,5].

We describe our experience with SIS in optimizing the outcome in nephron-sparing surgery.

## Methods

Between 08/03 and 10/06, all patients received a SIS to secure and apply compression to the defect following an open nephron-sparing surgery by two surgeons (JS, RP).

The patients were placed in a supine position. The retroperitoneal space was exposed by placing a pillow under the flank. The skin was incised transversally from the tip of the 11^th ^rib medially towards the epigastrium. The retroperitoneum was exposed and the Gerota's fascia was opened. The peritoneal sac was pushed medially. This maneuver exposed the aorta or the inferior caval vein as leading structure for further preparation. The kidney was freed from the perirenal fat. The fat overlying the tumor was sent for pathological examination. The renal artery and vein were identified and isolated with vessel loops. The renal capsule around the tumor was incised with a safety margin around the tumor. The tumor was bluntly dissected and excised with a margin of normal tissue using scissors or a Leriche dissector.

In patients with warm ischemia or cold perfusion, the patient received intravenous mannitol and furosemid prior to clamping to ensure adequate diuresis and to scavenge free radicals to decrease reperfusion injury. The renal artery and vessels were temporarily occluded with bulldog clamps or vessel clamps. In cases with central or large tumors where the anticipated time of warm ischemia could exceed 30 min, we performed an in-situ cold perfusion using Bretschneider solution (Composition: Sodium, Potassium, Magnesium, Chloride, Tryptophan, Potassium-α-ketoglutarate, Histidine-hydrochloride and Mannitol) as previously reported [6]. Briefly, after clamping of the renal artery, a longitudinal arteriotomy incision was made with a scalpel and extended with Pott's scissors. A 10 to 12 Ch. perfusion catheter was used to apply the solution. The renal vein was clamped and a longitudinal incision was made with a scalpel, or alternatively the left gonadal vein was divided. Immediately after these steps began the perfusion of the kidney with a hydrostatic pressure of 55 to 75 cm water. Continuous perfusion was maintained throughout the entire ischemia time. Following resection of the tumor and application of the SIS (see below), the vessels were closed with interrupted 6-zero polypropylene vascular sutures.

**Figure 1:**
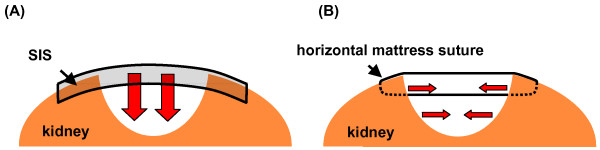
Comparison of the direction of the compression of the defect with SIS (A) and horizontal mattress sutures (B).

**Figure 2:**
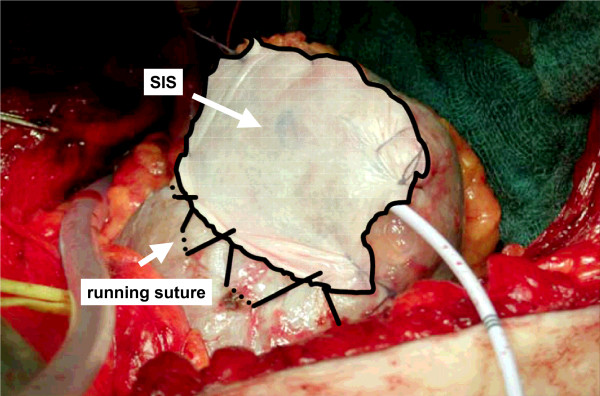
Scheme of the fixation of the SIS with a running suture, mimicking the fixation of a drumhead.

Visible blood vessels within the renal defect were ligated. The collecting system was closed with chromic sutures. For greater defects in the collecting system, a pyelostomy catheter was inserted. A haemostyptic agent [(FloSeal^® ^(Baxter, Deerfield, IL, USA), TachoSil^® ^(Nycomed, Unterschleissheim, Germany), or TachoComb^® ^(Nycomed, Unterschleissheim, Germany)] was transferred into the defect and the renal capsule was slightly approximated with interrupted sutures. The multilayered hydrated SIS was cut into shape. In the first 16 cases the SIS was fixed over the parenchymal defect using figure-of-eight chromic sutures. Care was taken to stitch the renal surface superficially to avoid parenchymal scarring caused by deep sutures. The direction of compression to the renal defect is different from the one applied by horizontal mattress sutures (Fig. 1). In the following 7 cases we used a running suture. In the remaining patients the hydrated SIS was tightened over the renal defect with a running suture mimicking the fixation of a drumhead (Fig. 2). A percutaneous drainage was placed to monitor bleeding and urinary leakage.

## Results

**Figure 3:**
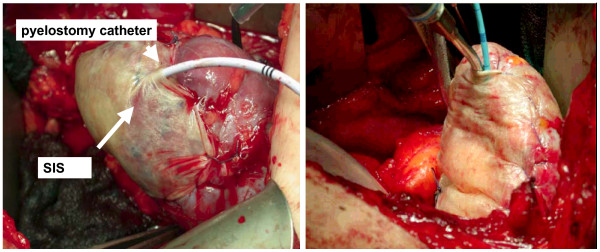
Surgisis^® ^Soft Tissue Graft (SIS technology) covering the renal defect, fixed with a running suture with a pyelostomy catheter in place.

Between 08/2003 and 10/2006 we performed 55 nephron-sparing procedures using SIS in 53 patients (median age: 59 yrs, range 29 – 79 yrs) (Fig. 3). The preoperative diagnostics revealed a solid lesion, suspicious for a malignant tumour or an angiomyolipoma in 48 (87.3%) and 7 lesions (12.7%), respectively. The indication for nephron-sparing surgery was elective in 38/53 (71.7%) patients. 6/53 patients (5.7%) had a single kidney, 3/53 patients (2.8%) had multiple renal tumors, 5/53 patients (4.7%) revealed an impaired renal function and 1/53 patient (0.9%) pts. had a tumor in the transplanted kidney.

According to the tumor location within the kidney and the tumor size 23/55 (41.8%), lesions were operated with in-situ cold perfusion. 18/55 (32.7%) and 14/55 (25.5%) of the tumors were resected without hilar control and with warm ischemia, respectively. Generally, it took 5 – 10 min to fix the SIS. The mean time of cold perfusion and warm ischemia was 69 min (range 37 – 145 min) and 18 min (5 – 29 min). The median operating time was 186 min (range 90 – 260 min) and the median blood loss was 730 cc (range 100 – 2500 cc). Perioperative (intraoperative or immediate postoperative) blood transfusion was required in 16 (29.1%) procedures.

The median tumor size was 4.5 cm (range 1.3 to 13 cm). The final pathology revealed a clear cell carcinoma in 39 cases (70.9%), a papillary carcinoma in 6 patients (10.9%), a papillary adenoma in 1 (1.8%), an oncocytoma in 2 (3.6%) and an angiomyolipoma in 7 cases (12.7%), respectively. All the tumors were resected in sano with negative margin status.

**Figure 4:**
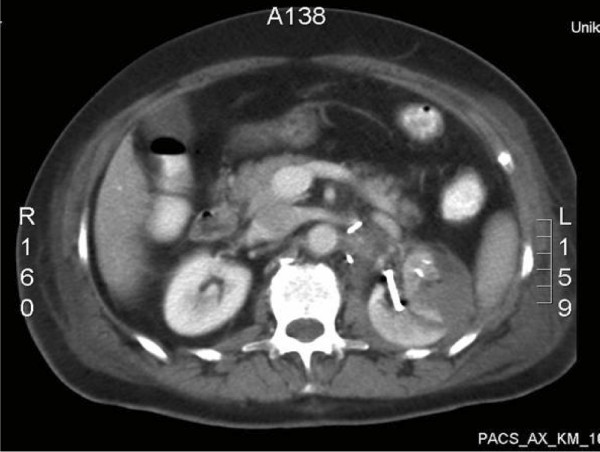
**CT scan of a patient with bleeding from the tumor bed.** The SIS secured the defect and stopped the bleeding by compressing the defect.

Concerning the postoperative complications, one patient (0.9%) died from a cerebral ischemia after surgery. One patient (0.9%) underwent relaparotomy due to postoperative bleeding from a failed suture of the venotomy following in-situ cold perfusion immediately after the primary surgical procedure. Minor continuous bleeding from a segmental renal artery following resection of an angiomyolipoma required interventional coiling in one further patient (0.9%) on postoperative day 3. Marked haematuria was the main symptom in this patient. In one patient (0.9%) a postoperative urinary leakage from the collecting system was noticeable. After the insertion of a double-J-catheter on day 4 following the nephron-sparing surgery, the fistula closed without any further intervention and without urinoma formation. In one patient (0.9%) we performed a postoperative CT scan to evaluate prolonged haematuria on postoperative day 6. Only blood clots were present in the resection area. However, no further active bleeding was detected in the CT scan. In this patient, the SIS membrane secured the defect and stopped the bleeding by compressing the defect (Fig. 4). In no patient was an open operative revision due to postoperative haemorrhage from the surgical site indicated. Furthermore, no transfusions were necessary to substitute persistent blood loss from drains postoperatively.

## Discussion

On a standard basis, nephron-sparing surgery has been used in patients with a renal cell carcinoma having a solitary kidney, bilateral synchronous tumors or a systemic disease that could potentially jeopardize renal function [7]. However, due to the recently published promising cancer-free survival rates of patients undergoing nephron-sparing surgery for tumors less than 4 cm, this approach gains widespread acceptance in patients with a normally functioning contralateral kidney [8-10]. Furthermore, Leibovich et al. [11] demonstrated that even patients with renal cell carcinoma from 4 to 7 cm treated with nephron-sparing surgery had an excellent cancer-specific and metastases-free survival when the tumors had an exophytic growth pattern and presented with minimal risk of invasion of the collecting system. These promising oncological results, combined with advances in renal imaging and improved surgical techniques, led to an increase of nephron-sparing procedures.

The critical steps during nephron-sparing surgery are the resection of the tumor with an adequate margin of benign tissue around the tumor, as well as a sufficient haemostasis and closure of the collecting system. The surgical technique applied depends on the size and location of the renal tumor.

In little peripheral renal masses, haemostasis may be achieved after tumor resection using coagulation and a haemostyptic agent without any sutures. Finley et al. [12] reported on a series of 15 patients who underwent a sutureless haemostasis after excision of small renal masses (range 1.1 to 3.5 cm) using a fibrin glue – oxidized cellulose sandwich. Otherwise, the mainstay of haemostasis has to be accomplished by compressing the tissue around the renal defect. Usually, for this purpose the renal capsule is sutured with horizontal mattress sutures over the length of the defect [13]. There is a theoretical possibility of loss of functional kidney tissue due to scarring caused by the suture line passing through the renal capsule and parenchyma, even though to date no experimental data exist supporting this hypothesis. The maximum tensile force that may be applied onto the sutures is limited by the tensile strength of the renal capsule and parenchyma. The tearing of the suture through the capsule is of major concern. Therefore, alternative methods to buttress the closure of the renal parenchyma using exogenous material like Gore-Tex [13] or Teflon [14] have been investigated. The exogenous materials prevent the tearing of the sutures through the renal parenchyma, leading to a tighter, more haemostatic and watertight closure of the defect [13].

In the case of larger defects, the renal parenchyma has to be folded to close the defect with sutures. Therefore, the closure is hard to achieve and associated with a greater risk of complications [2]. In these cases, the use of exogenous materials like perirenal fat or oxidized cellulose was propagated to cover the defect [15,16]. Wainstein et al. [17] successfully used a polyglycolic acid mesh in three patients to obtain a secure closure of a renal defect [17]. In a recent study, O'Connor et al. [18] described the use of SIS to cover the defect in 24 partial nephrectomies. In their study, haemostasis was achieved by excising the defect with a harmonic scalpel, using coagulation of the parenchyma with an argon beam, ligation of visible vessels and closing the collecting system with interrupted, chromic sutures. The SIS was placed over the defect with several figure-of-eight chromic sutures.

Compared to the study by O'Connor et al. [18], we optimized the haemostyptic procedure. To minimize the loss of functional kidney tissue, we used neither a harmonic scalpel to excise the tumor nor the argon beamer to coagulate the parenchyma. The adverse effect of coagulation on the remaining renal parenchyma was demonstrated in the study by Murphy et al. [19]. Microscopic changes of the renal parenchyma caused by the applied heat were noted as deep as 1 cm below the surface area. To achieve haemostasis, we ligated visible vessels and applied a haemostyptic agent. Furthermore, we only slightly approximated the edges of the kidney without applying tension on the renal capsule. Finally, we fixed the hydrated SIS more tightly over the renal defect with a running suture mimicking the fixation of a drumhead. This led to a uniform tension on the renal defect (Fig. 1).

The intraoperative blood loss (median 730 cc) and transfusion rate (29.1%) in the present study is comparable to the literature [20-23]. Furthermore, the reported incidence of postoperative bleeding complications and urinary fistulas following nephron-sparing surgery is 0 – 7.9% and 1 – 10.1%, respectively (see Table 1) [3,9,20,22-26]. However, when comparing the data in the literature, it is mandatory to consider the diameter of the renal lesions. Due to the broadening of the indication for elective nephron-sparing surgery in recent years, only few studies exist reporting complication rates in series with larger tumors such as we present in our study. Both the postoperative bleeding and urinary leakage rates were 1.8%. These results are superior to recent studies with comparable tumor size, reporting bleeding rates of 2.9% and urinary leakage rates of 5.8% and 10.1% [20,24].

**Table 1:** Reported major peri- and postoperative surgical complications after nephron-sparing surgery for solid renal lesions

Reference	Pat. (n)	Tumor size (cm) [range]	Estimated blood loss (cc) [range]	Transfusion rate (%)	Postoperative bleeding n (%)	Postoperative urinary leackage n (%)	Open revision n (%)
Polascik TJ et al 1995 [3]	67	NA	NA	NA	0	6 (8.9)	0
Lerner SE et al 1996 [9]	185	NA	NA	NA	0	4 (2.1)	3 (1.6)
Margulis V et al 2007 [20]	34	5.2*	975*	28.1	1 (2.9)	2 (5.8)	1 (2.9)
Gill IS et al 2007 [21]	1029	3.5* [0.6–7.0]	376* [10–3300]	5.1	NA	NA	NA
Duque JLF et al 1998 [22]	66	3.5* [1.1–12.0]	836* [100–3200]	56	3 (4.5)	6 (9.1%)	0
Schiff JD et al 2005 [23]	59	3.4*	363*	NA	0	1 (1.7)	0
Becker F et al 2006 [24]	69	5.3* [4.1–10.0]	NA	NA	2 (2.9)	7 (10.1)	2 (2.9)
Belldegrun A et al 1999 [25]	146	3.6* [2.0–9.0]	NA	NA	3 (2.1)	2 (1.4)	3 (2.1)
Van Poppel H et al 1998 [26]	76	3.3* [0.9–15]	NA	NA	6 (7.9)	1 (1.3)	2 (2.6)
present series	55	4.5+ [1.3–13]	730+ [100–2500]	29.1	1 (1.8)	1 (1.8)	0

*mean, +median, # definitions of postoperative bleeding varied among series.

## Conclusion

In expanding the indications for nephron-sparing surgery, there is a need to establish reliable techniques to excise the tumor and prevent postoperative complications even in larger tumors. In this context, SIS has proven to be a highly effective and easy-to-use instrument for preventing postoperative bleeding and urinary fistula complications.

## Abbreviations

SIS: small-intestine submucosa.

## Competing interests

The authors declare that they have no competing interests.

## Authors' contributions

JS made substantial contributions to conception and design, performed a significant part of the operations, have written the manuscript. RP made substantial contributions to conception and design, performed the most operations. MM made a substantially effort in collecting the data material. REH revised the manuscript critically and made a substantially intellectual input. GB was involved in drafting the manuscript and revising it critically for important intellectual content. All authors read and approved the final manuscript.

## Pre-publication history

The pre-publication history for this paper can be accessed here:


